# Possible Nonneurological Health Benefits of Ketogenic Diet: Review of Scientific Reports over the Past Decade

**DOI:** 10.1155/2022/7531518

**Published:** 2022-05-27

**Authors:** Katarzyna Daria Gołąbek, Bożena Regulska-Ilow

**Affiliations:** Wroclaw Medical University, Department of Dietetics, Wrocław, Poland

## Abstract

The ketogenic diet (KD) has been used since the 1920s as a therapy for drug-resistant epilepsy. Due to the beneficial effects of this diet on the nervous system and the proposed multifaceted effects of ketones on health and disease, researchers have evaluated its use in other nonneurological conditions. The objective of this review was to analyze the most recent papers, which is why meta-analyses were used in which 75% of the studies were from 2012 to 2022. Authors also cited single studies from the last decade that lasted longer than 12 months to assess the long-term benefits of KD. Reports from the past decade have highlighted several significant areas regarding the impact of KD. One of these is the use of very low-calorie ketogenic diet (VLCKD) as an effective possibly safe and patient-motivating component of a long-term weight loss plan. Reports on the positive influence of KD on the health of obese individuals, and the possible resulting validity of its use, should be verified by patients' physical activity levels. A significant number of studies from the last decade evaluate the effect of KD on improving the health of individuals with type 2 diabetes as an effective tool in lowering glycated hemoglobin (Hb1Ac) and required doses of hypoglycemic drugs. The long-term studies indicate a possible beneficial effect of KD on cardiovascular function due to improvement lipid profile, changes in apolipoprotein (Apo) A1, adiponectin, and intercellular adhesion molecule-1 (ICAM-1).

## 1. Introduction

A ketogenic diet (KD) is a very low-carbohydrate diet. The reduced supply of carbohydrates decreases glucose availability in the body, which triggers a change in cellular energy utilization [1]. This promotes lipolysis and the utilization of free fatty acids rather than glucose for energy metabolism. In turn, limited glucose availability leads to the production of ketones (e.g., acetoacetate, *β*-hydroxybutyrate, and acetone) as alternative energy sources [2].

The KD has been used since the 1920s as a therapy for drug-resistant epilepsy [3]. In the last two decades, the popularity of this dietary strategy has grown. Between 2004 and 2019, the KD was the fifth diet that generated significant interest online [4].

Emerging evidence suggest that ketone bodies are not only energy substrates but exert pleiotropic effects on mitochondrial functioning, function of signaling mediators, and contribute to endogenous antioxidant defenses [5–7]. Due to the proposed multifaceted effects of ketone bodies, multiple studies have examined the possible benefits, efficacy, and safety of the KD on nervous system functioning and as a possible intervention across multiple diseases [8].

In KD, the proportion of energy obtained from carbohydrates oscillates between 14% and 20%, which is mostly less than 50 grams (*g*) of carbohydrates (CHO) per day (d) [9–13]. In the beginning of keto-adaptation, these values are frequently lowered to approximately 25 g/d [11, 12, 14, 15].

Over the course of two days to a week on average, very low carbohydrate intake causes an increase in serum levels of ketone bodies [14, 16]. In general, studies show that a concentration higher than 0.5 mM/L is indicative of stable ketosis, although others report a higher value (0.8–1 mM/L). In turn, this stabilization is linked to the resolution of initial adverse effects including nausea, malaise, dizziness, polyuria, low mood, and constipation, often termed as “keto flu” [16–18]. Recent reports indicate that the level of ketone production and the body's response to ketosis may be genetically determined [19].

This narrative review focuses on research papers from the last decade evaluating the use of ketogenic diet in diseases unrelated to the nervous system. To analyze the most recent papers, meta-analyses were used in which 75% of the studies were from 2012 to 2022. Many studies indicate short-term beneficial effects of KD, but to complement them, the following article also cites single studies from the last decade that lasted longer than 12 months to assess the long-term benefits of KD.

## 2. The Use of the Ketogenic Diet in Individuals with Excessive Body Weight

For many years, low-carbohydrate diets such as the Atkins diet and the KD have been popular tools used for weight loss. In the last decade, many researchers have been evaluating the validity of the very low-calorie ketogenic diet (VLCKD) for weight loss by obese individuals. Assuming, in different variations depending on the authors' implementation, a 3-stage protocol in which stage 1 implements <600–800 kcal/d and about 30–50 g CHO, followed by the introduction of more calories and carbohydrates (about 800–1500 kcal/d of the so-called dietary re-education -stage 2), and then a diet of about 1500–2250 kcal/d.

A meta-analysis of 12 studies by Castellan et al. [20] evaluated the beneficial short- and long-term effects of VLCKD in overweight participants. Maintaining the ketosis stage for 4 weeks was associated with an average loss of 10 kg (*I*^2^ = 6%), and about 15.6 kg (*I*^2^ = 37%) between 4 and 12 weeks (6 studies). It was estimated that approximately (approx.) 66% of the weight lost was adipose tissue. The obtained anthropometric changes were stable in follow-ups of all studies, with the shortest lasting 3 weeks and the longest about 2 years. In this meta-analysis, both from the beginning of the study to 4 weeks and from 4 to 12 weeks, significant reductions in body mass index (BMI) values (−4.2; *I*^2^ = 77%; −6.2 kg/m2; *I*^2^ = 73%) and waist circumference (WC) values (−9.7; *I*^2^ = 67%; −15.6 cm; *I*^2^ = 76%) were reported along with the weight loss. Higher values were obtained with longer maintenance of ketosis. In addition, use of this dietary strategy was associated with reductions in glycated hemoglobin (HbA1c), total cholesterol (TC), triglycerides (TG), aspartate transaminase (AST), alanine aminotransferase (ALT), gamma-glutamyl transferase (GGT), systolic blood pressure (SBP), and diastolic blood pressure (DBP). Changes in low-density lipoprotein cholesterol (LDL-c), high-density lipoprotein cholesterol (HDL-c), serum creatinine, serum uric acid, and serum potassium were not observed.

Based on the cited meta-analysis, it is worth noting the 2 long-term (>12 m) studies used in it by Moreno et al. [21, 22]. The first, conducted in 2014 [21] on a group of 53 participants, compared the VLCKD protocol with a low-calorie diet (LCD) (about 90% total metabolic expenditure) containing 45–55% of carbohydrates. In the study, the ketosis stage lasted about 30–45 days. After 12 months of intervention, significantly higher weight loss (19.9 ± 12.3 vs 7.0 ± 5.6 kg; *p* < 0.0001) and thus BMI values were observed in VLCKD participants compared to LCD. Changes in lean body mass were not assessed in the VLCKD group. However, this method was also associated with a higher incidence of side effects like fatigue, headache, muscle weakness, constipation, hyperuricemia, and nausea compared to LCD. Most participants observed their resolution after a few weeks. However, after one year of intervention, 18.5% of participants still reported constipation, 7.45% hair loss, and 3.7% fatigue. During the study, changes in body weight and studied biochemical parameters were observed according to the stage of diet, carbohydrate content, and caloric value. In addition to anthropometric changes, significant reductions in HbA1c (5.50 vs 5.49%) and TC (207.2 vs 193.2 mg/dL) were observed in the VLCKD group at 12 month of the study.

A 2016 study by the same author [22] also compared the impact of the VLCKD protocol, which included a 2-month ketosis phase, with LCD over a 2-year period. The VLCKD group was evaluated to have significantly higher weight loss (12.5 vs 5.2 kg; *p* < 0.001), reduction in BMI (4.4 vs 1.9 kg/m^2^; *p* < 0.001), and fat loss (8.8 vs 3.8 kg; *p* < 0.001) compared to the LCD group. Moreover, the VLCKD group lost significantly more visceral fat−666 vs 200 *g* (*p* < 0.001). After 2 years, significantly more (54%) of VLCKD group participants lost and maintained 10% weight loss compared to LCD (13%; *p* < 0.001).

The positive anthropometric changes described in previous papers were also confirmed in a meta-analysis of European studies [23] comparing the effect of VLCKD and other dietary strategies such as very low-calorie diet (VLCD), LCD, or Mediterranean diet with calorie restriction. Differently to the work of Moreno et al. [21, 22] and Castellana et al. [20] in which no changes in fat free mass (FFM) were noticed, in this meta-analysis [23] a decrease in FFM of −2.96 kg (*I*^2^ = 0%) was observed in the groups using VLCKD. However, this result was not significantly different from other assessed interventions. The use of VLCKD improved the biochemical profile of the participants; however, the reduction in the values of three parameters: homeostatic model assessment for insulin resistance (HOMA-IR) (−1,36; *I*^2^ = 98%), TC (−7,13 mg/dL; *I*^2^ = 51%), TG ( ~−29,90 mg/dL; *I*^2^ = 89%) was significantly higher in the VLCKD group compared to other dietary strategies.

In the last decade, researchers have been combining or contrasting the benefits gained from the ketogenic diet with the effects of the Mediterranean diet that has been recommended for years. The study by Petricone et al. [24] compared the use of VLCKD and a calorie-restricted Mediterranean diet (Total Daily Energy Expenditure (TDEE) reduced approx. 500 kcal; 55–60% CHO) in two 28-person groups over 12 months. Significant reductions were observed in WC (119.1 ± 22.9 vs 95.0 ± 17.4 cm), HbA1c (6.1 ± 1.4 vs 5.2 ± 0.15%), HOMA-IR values (7.3 ± 0.7 vs 2.6 ± 0.2), fasting insulin (28.0 ± 16.7 vs, 10.9 ± 4.9 *µ*U/L), TG (151.3 ± 50.0 vs 72.3 ± 29.6 mg/dL), C-reactive protein (CRP) (4.5 ± 2.6 vs 1.8 ± 0.8 mg/dL) in the VLCKD group compared to the beginning of the study. It is worth noting that an increase in 25 (OH)D status (18.4 ± 5.9 vs 29.3 ± 6.8 ng/mL) was observed in the blood of participants of this group. Participants following the Mediterranean diet showed no significant improvement in anthropometric and 25 (OH)D parameters, while significant biochemical changes were observed in fasting Glc, HbA1c, fasting insulin, HOMA, TG, and CRP.

In another study [25], a 20-day period of VLCKD (30 *g* CHO/d, 36% protein, 52% fat; approx. 976 ± 118 kcal) starting the study was combined with a 20-day low-carbohydrate nonketogenic diet (LCNKD) (25% CHO, 31% protein, 44% fat; approx. 1111 ± 65 kcal) and then a 4-month norm caloric Mediterranean diet (58% CHO, 15% protein, 27% fat; approx. 1800 ± 100 kcal). The entire cycle was then repeated extending the Mediterranean diet period to 6 months. In the study, both periods of VLCKD were associated with ketosis and contributed to significantly higher decreases in body weight (100.7 ± 16.54 vs 84.59 ± 9.71 kg) and body fat percentage (43.44 ± 6.34 vs 33.63 ± 7.6%) compared to the LCNKD or Mediterranean diet stages, during which no significant changes were assessed. Results were maintained until the end of the study, similarly to positive changes in TC, LDL-c, and fasting Glc, whose significant decrease was observed after 1 period of VLCKD and LCNKD.

Such trends were also observed in another population [26]. In addition, no significant changes in estimated glomerular filtration rate (eGFR), creatinine levels, or microalbuminuria were observed during the 12-month study with VLCKD. During the study, a subtle but significant improvement in uricemia was reported following the end of the ketogenic period. Changes in potassium, sodium, and magnesium status were not observed. Only calcium concentrations decreased slightly during the first 30 days of the study and then increased between days 30 and 90.

The protocols used in the above meta-analyses and studies that included VLCKD diet periods had a very high restriction of caloric supply. Therefore, it is difficult to conclude whether ketosis, caloric deficit, or their synergistic effects were the key to achieving the described benefits. Maintaining such a caloric regimen is a great challenge for participants, hence the assessed periods of ketogenic diet lasted 2 months at most. However, it is worth noting that after the restrictive periods, the caloric values from the VLCKD groups equaled other dietary strategies, remaining more effective long-term. Despite this, reports from this decade still do not provide a complete answer regarding the safety of long-term KD use. In most of them, carbohydrate content increases again over longer periods of time.

All the cited works support the effectiveness of introducing even short periods of KD as a tool for effective weight loss possible to be sustained long term. Even its short-term application is a viable achievement for patients that can increase motivation and traction to improve biochemical parameters of glycemia and lipid profile.

Apart from optimistic reports of using VLCKD, researchers also point to less calorically restrictive eating plans as positive tools for weight loss. For instance, in a long-term study involving six months of controlled dietary intervention and an 18-month follow-up, a nutrigenomic tailored and low glycemic index diet was found to be more effective in attaining and maintaining weight loss compared to the KD. Furthermore, it contributed to greater reductions in TC and fasting Glc and an increase in HDL-c compared to the results obtained in the KD [27].

## 3. The Ketogenic Diet Combined with Physical Activity for Weight Loss

Optimistic reports on the achieved effects of using VLCKD among overweight individuals without added physical activity are worth contrasting with the results of the meta-analysis by Asthary-Larky et al. [28] regarding the effects of less restrictive KDs combined with resistance training. Based on 13 studies (92.3% from the last decade) lasting maximum 3–4 months, the authors concluded that compared to diets providing 40–50% of carbohydrates and KD was associated with significantly greater reductions in body weight (*I*^2^ = 18, 1%), fat mass (*I*^2^ = 62, 4%), and percentage of body fat (*I*^2^ = 79, 8%), as well as BMI (*I*^2^ = 68, 9%). However, the same analysis (similarly to [23]) showed a negative effect of KD on FFM (WMD = −1.26 kg; 95% CI: −1.82, −0.70; *I*^2^ = 22, 7%; *p* < 0.001). This may be because 10 of the 13 studies analyzed the outcomes of highly physically active individuals, both nonelite and elite athletes, in contrast to previously cited studies involving inactive obese individuals. Based on further subgroup analysis, it was found that KD had no significant effect on FFM in overweight participants.

Another meta-analysis [29] of 7 studies (the longest was 6 months) compared the benefits gained from KD (<50 CHO g/d) combined with physical activity (CrossFit, cycling, progressive resistance training, combination of aerobics, and resistance exercise) and among overweight participants with regular diets (contains approx. 40–55% CHO) who are increasing their physical activity. In the reviewed papers, the period of ketosis lasted an average of 9.2 weeks (min. 4; max. 24). The only significant favorable changes of anthropometric indices in the KD group were related to a decrease in WC (2 trials; *I*^2^ = 0%) compared to the control groups. A comparison of four studies showed no significant differences between groups for VO_2_ max. However, in both studies the introduction of regular activity was associated with a significant increase in performance. Significantly higher decreases in TG (4 trials; *I*^2^ = 0%) were observed among KD users; however, more favorable changes were not observed in fasting Glc, LDL-c, and TC.

It appears that the participant's willingness to increase physical activity may prove to be a key element in selecting an appropriate dietary strategy for weight loss. The greater efficacy of KD in inactive individuals with excessive body weight is not as clearly confirmed in those who increase their level of physical activity. Diets that are easier to maintain, less restrictive in carbohydrate content, and caloric value, seem to be an equally valid choice, perhaps allowing for better maintenance of muscle mass. The selection of the appropriate type of physical activity predisposing to greater weight loss in combination with appropriate dietary strategies, including KD, seems to be an interesting research question.

## 4. The Use of the Ketogenic Diet in Patients with Type 2 Diabetes

Because of the limited intake of glucose sources and other simple sugars to regulate glycemia, low-carbohydrate diets are a common choice for patients with type 2 diabetes (T2D). Over the past decade, researchers have continued to evaluate the impact of KD use on required doses of hypoglycemic medications and observed additional lifestyle-related benefits of this dietary strategy.

A meta-analysis of 12 studies ranging from 1 to 52 weeks [30] involving overweight participants evaluated the effect of KD (max. 50 g CHO/d) on biochemical parameters associated with T2D. Compared to the beginning of the intervention, a reduction (95% CI) in Glc of 1.29 mmol/L (10 trials; *I*^2^ = 68%) and a significant reduction in HbA1c by −1.07% (8 trials; *I*^2^ = 71%) percentage points were observed, which is considered an ideal pharmacotherapeutic effect. Eight of these studies also evaluated TG reduction of 0.33 mmol/L (*I*^2^ = 67%), LDL-c of 0.05 (*I*^2^ = 71%), and HDL-c increase of 0.14 mmol/L (*I*^2^ = 78%). Similar to the studies with metabolically uncomplicated obese participants, this group also showed a significant decrease in body weight (8.66 kg; *I*^2^ = 92%), decrease in WC (−9.17 cm; *I*^2^ = 0%), and BMI values (3.13 kg/m^2;^*I*^2^ = 28%).

Another meta-analysis by Alarim et al. [31] observed more beneficial changes of using KD, mainly VLCKD (about 20–50 g CHO/d), compared to the work of Yuan et al. [30]. However, this meta-analysis included three times fewer more heterogeneous studies. The authors evaluated significantly higher weight loss (*I*^2^ = 81%), decreased BMI (*I*^2^ = 97%), decreased fasting Glc (*I*^2^ = 98%), HbA1c (*I*^2^ = 98%), TG (*I*^2^ = 95%), and TC (*I*^2^ = 98%) in the KD group compared to control groups following calorie-restricted and/or low glycemic index diets. However, despite increased fat intake, no increase in LDL-c was observed, and a significant increase in HDL-c (*I*^2^ = 97%) was additionally reported.

The results of meta-analyses are supported by long-term studies. One of them encompassed 16 participants [32] with excess body weight characterized by HbA1c >6% that used KD (20–50 g assimilable CHO/d) in combination with mindful eating and positive attitude techniques, improved sleep hygiene and physical activity to achieve ketosis (0.5–3 mmol/L), and improved health. This 12-month intervention reduced HbA1c from 6.6% to 6.1%, a mean loss of 7.9 kg, and a reduction in hypoglycemic medication doses. All the obtained results were significantly higher compared to the group following a low-fat diet (LFD) (45–50% CHO, TDEE reduced approx. 500 kcal). It is worth noting that the caloric content of both diets was not statistically different.

The effect of reducing the required doses of hypoglycemic drugs was also observed in the work of Tay et al. [33,34] at both 12- and 24-month periods. Although the effects of KD (approx. 70% of TDEE−1700 kcal, 14% CHO, 28% protein, and 58% fat) compared to a more isocaloric high-carbohydrate diet (approx. 70% of TDEE −1700 kcal, 53% CHO, 17% protein, and 30% fat) were not significantly different with respect to body weight (9% of body weight lost in both groups), decreases in blood pressure, body fat percentage, LDL-c, HbA1c, and fasting Glc. However, a low-carbohydrate diet was associated with more stable Glc levels throughout the day and less frequent hyperglycemic episodes after 52 weeks. The low-carbohydrate group also showed a greater reduction in TG after 52 weeks and 104 weeks. HDL-c was maintained after 104 weeks. In the carbohydrate-restricted group, a 20% reduction in the required doses of hypoglycemic medications was observed in a higher percentage of participants (52%; 67%) compared to the high-carbohydrate group (21%; 32%) after 52 weeks and 104 weeks. What is more, changes in non-HDL-c, TC, LDL-c, blood pressure, and CRP did not differ between groups. Endothelial function did not change in either group after 2 years.

Patients with diabetes often choose the macronutrient content and duration of low-carbohydrate diets by themselves. Webster et al. [35] in their study evaluated the effects of a low-carbohydrate high-fat (LCHF) diet in a group of participants following it for at least 6 months to a maximum of 6 years. Six months of LCHF use was the minimum period to be included in the study. During the 15-month study, participants consumed an average of 61 g of CHO/d. This intervention contributed to a decrease in median HbA1c from 7.5% to 5.9% and a reduction in hypoglycemic medication doses. Remission and discontinuation of medications as a result of the diet was achieved by 29% of participants (7 of 22). The majority of them reported reduced hunger, frequency of meals, snacking, and a desire to eat sweet foods while using KD.

Studies from the last decade suggest that in addition to improving glycemic parameters, KD may also improve other health components. The study executed by Vilar-Gomez et al. [36] observed its possible beneficial effect on hepatic steatosis, as 12 months of using KD resulted in reduced values of indices assessing hepatic steatosis and fibrosis risk. Such changes were not observed in diabetic patients receiving standard treatment. Improvements in diabetes-related parameters were associated with improvements in ALT levels in the KD group.

In addition, Moricione et al. [37] observed that a 3-month period of best adherence to KD decreased the incidence of emotional and uncontrolled eating in the T2D group, whereas incidents of uncontrolled eating increased in the comparison group using LCD. After 12 months, the VLCKD group showed improvement in physical and psychological quality of life, with participants showing higher levels of satisfaction with weight loss, well-being, and dietary management.

Additionally, other authors [38] reported improved overall sleep quality in patients with prediabetes and diabetes after 12 months of using KD as opposed to those receiving standard treatment. It is noteworthy that sleep quality significantly improved among poor sleepers during the intervention. Interestingly, increased ketone levels correlated with better sleep quality in the prediabetic group.

Both meta-analyses and long-term studies point to possible beneficial effects of KD in T2D other than weight loss and improved body composition. In particular, it concerns the lowering of HbA1c levels and decreasing the required doses of hypoglycemic drugs. It is worth emphasizing that this effect is also visible in comparison to isocaloric diets with other fat and carbohydrate contents.

## 5. The Use of the Ketogenic Diet in Individuals at Increased Risk for Cardiovascular Disease

Inflammation and oxidative stress are important pathogenic mechanisms implicated in cardiovascular disease (CVD) [39]. The KD, through lowered carbohydrate intake and better regulation of glycemic control, coupled with the possible anti-inflammatory effects of ketone bodies themselves might additively contribute to the prevention of CVD [5–7].

In addition to the observed beneficial changes in lipid profile resulting from the use of KD such as the observed reduction in TG [20, 21, 23, 24, 29–31, 34], TC [20, 22, 23, 25, 31], improvement in LDL-c [25, 30], and maintenance or increase in HDL-c [30, 31, 33, 34], researchers observe a possible long-term additional effect of KD on CVD.

In a nonrandomized study involving 226 patients with T2D, after 52 weeks of using KD, Bhanpuri et al. [40] reported a significant decrease in TG, TG/HDL-c ratio, and blood pressure, as well as an increase in apolipoprotein (Apo) A1, Apo B/Apo A1 ratio, LDL-c, and HDL-c compared to the beginning of the study.

In another group of participants not complicated by T2D and CVD, lowering CHO intake <40 g/d for 12 months did not alter proinflammatory interleukin (IL)-6, IL-8, and tumor necrosis factor (TNF)-*α* levels. However, it induced a significant increase in adiponectin. After 12 months, no significant changes in resistin, leptin, and E-selectin concentrations were observed. The concentration of intercellular adhesion molecule –1 (ICAM-1), which promotes endothelial damage and atherosclerosis, did not increase in the KD group, in contrast to the LF diet group. The analyzed changes in adiponectin and ICAM-1 concentrations in 53.6% and 69.5% were not dependent on the level of weight loss [41].

The effect of KD on CVD requires more specific studies evaluating more factors that promote prevention or occurrence of CVD. The cited long-term studies from the last decade indicate a possible beneficial effect of KD on cardiovascular function due to changes in ApoA1, adiponectin, and ICAM-1. It is worth noting that KD also improves the lipid profile. These positive reports need to be verified by more specific, long-term studies, and meta-analyses, which are still scarce in the available current literature.

## 6. Limitations

Finally, it is worth emphasizing the limitations of this narrative review. The chosen papers indicate important current areas of research on the impact of KD, showing current trends and additions to data from previous decades. However, the work does not illustrate all available knowledge on the subject and does not represent a complete compendium on KD.

Another limitation is heterogeneity between studies used in meta-analyses. For most authors, the acceptable heterogeneity was determined by I^2^ cut points <50% [20, 28–31], except for the study by Mascoguri G et al. [23] where a limit of <60% was set. Most of the obtained results, apart from the weight loss [20, 23, 28, 29], BMI [23, 28–30], and FFM [20, 23] were characterized as medium ( *I*^2^>50%–75%) or highly (*I*^2^>75%) heterogeneous [42]. In this aspect, it is worth emphasizing the low heterogeneity (*I*^2^ <25%) between studies in meta-analysis by Lee H et al. [29] (most results obtain *I*^2^ = 0), in contrast to Alairm et al. [31] (most results exceed *I*^2^>95%). It is important to carefully reach conclusions from cited meta-analyses also in the perspective of heterogeneity, not to overestimate positive results.

## 7. Conclusions

Reports from the past decade have highlighted several significant areas regarding the impact and following KD. One of these is the use of VLCKD as an effective and patient-motivating component of a long-term weight loss plan. In the cited studies, short periods of very low-calorie KD were effective and their effects were maintained even after increasing calories and equating them to comparable dietary strategies such as LF, reduced-calorie Mediterranean diet, VLCD, and LCD. Current evidence confirms that positive anthropometric changes (weight loss, reduction of body fat, including visceral fat), as well as the beneficial effects on lipid profile (TG, TC, and LDL-c reduction) obtained during VLCKD can be sustained in the long term (12–24 months).

Reports on the positive influence of KD on the health of obese individuals and the possible resulting validity of its use should be verified by patients' physical activity levels. Studies from the last decade do not present such a clear message in terms of the beneficial use of KD by physically active individuals as in trials that do not include increased activity. Doubts about the introduction of KD are particularly related to maintaining muscle mass.

A significant number of studies from the last decade evaluate the effect of KD on improving the health of individuals with T2D. Based on current reports, it can be concluded that using KD is more effective in lowering Hb1Ac as well as the required doses of hypoglycemic drugs compared to other dietary strategies. This beneficial effect was also observed when the period of adherence to 50 g CHO/d intake was short and lasted up to 6 months. Moreover, interesting new research topics have emerged, such as the effect of KD on the level of hepatic steatosis, appetite regulation, and sleep quality among T2D patients.

However, despite its beneficial influence, KD can be complicated by noticeable side effects that can last up to 12 months. Using KD requires constant health monitoring as well as selecting proper macronutrients and hydration, due to the possible occurrence of disorders such as kidney stones. Their prevalence in adults using KD is about 7.9% [43].

Current reports still do not provide complete data to determine whether the long-term regimen of carbohydrate intake demonstrated over periods of 5, 10, and 15 years is safe. In most of the cited papers, the period of full ketosis and CHO restriction <50 g/d lasted less than 6 months.

There is still a lack of studies that comprehensively evaluate changes in health status in patients following KD. To prove its safety, several factors need to be explored, including the extent of weight loss, changes in lipid and glucose profiles/T2D, full blood counts, iron parameters, renal function, endocrine changes, gut microbiota, and micronutrient deficiencies.

## Data Availability

All publications used in the work are available in PubMed and online.
